# Retention of progenitor cell phenotype in otospheres from guinea pig and mouse cochlea

**DOI:** 10.1186/1479-5876-8-119

**Published:** 2010-11-18

**Authors:** Jeanne Oiticica, Luiz Carlos M Barboza-Junior, Ana Carla Batissoco, Karina Lezirovitz, Regina C Mingroni-Netto, Luciana A Haddad, Ricardo F Bento

**Affiliations:** 1 Department of Otolaryngology, Medical School, University of São Paulo, São Paulo, Brasil; 2 Department of Genetics and Evolutionary Biology, Institute of Biosciences, University of São Paulo, São Paulo, Brasil

## Abstract

**Background:**

Culturing otospheres from dissociated organ of Corti is an appropriate starting point aiming at the development of cell therapy for hair cell loss. Although guinea pigs have been widely used as an excellent experimental model for studying the biology of the inner ear, the mouse cochlea has been more suitable for yielding otospheres *in vitro*. The aim of this study was to compare conditions and outcomes of otosphere suspension cultures from dissociated organ of Corti of either mouse or guinea pig at postnatal day three (P3), and to evaluate the guinea pig as a potential cochlea donor for preclinical cell therapy.

**Methods:**

Organs of Corti were surgically isolated from P3 guinea pig or mouse cochlea, dissociated and cultivated under non-adherent conditions. Cultures were maintained in serum-free DMEM:F12 medium, supplemented with epidermal growth factor (EGF) plus either basic fibroblast growth factor (bFGF) or transforming growth factor alpha (TGFα). Immunofluorescence assays were conducted for phenotype characterization.

**Results:**

The TGFα group presented a number of spheres significantly higher than the bFGF group. Although mouse cultures yielded more cells per sphere than guinea pig cultures, sox2 and nestin distributed similarly in otosphere cells from both organisms. We present evidence that otospheres retain properties of inner ear progenitor cells such as self-renewal, proliferation, and differentiation into hair cells or supporting cells.

**Conclusions:**

Dissociated guinea pig cochlea produced otospheres *in vitro*, expressing sox2 and nestin similarly to mouse otospheres. Our data is supporting evidence for the presence of inner ear progenitor cells in the postnatal guinea pig. However, there is limited viability for these cells in neonatal guinea pig cochlea when compared to the differentiation potential observed for the mouse organ of Corti at the same developmental stage.

## Introduction

The sense of hearing, one of the five primary senses, is mediated through a complex sensory system that allows the perception and reaction to a huge variety of sound stimuli. Hearing makes feasible individual interaction with the environment and is essential for communication. Typically, the auditory system comprises a highly specialized sensory epithelium, the organ of Corti. It contains mechanosensory hair cells as the primary transducers of auditory stimuli, and supporting cells that provide a structural and physiological supporting epithelium. One end of hair cells interacts with physical inputs and transmits these signals to the neural circuits, linked to the opposite end of the cell by a synapsis[1]. Most types of congenital and acquired hearing loss arise from damage and irreversible loss of cochlear hair cells or their associated neurons[2].

A remarkable characteristic of highly differentiated and specialized mammalian cells, including cochlear sensory hair cells, is that after birth they are held in a post-mitotic state which contributes to their terminal differentiation and inability of repair[3]. A complex network of cyclin-dependent kinases and negative cell cycle regulators are involved in blocking cell cycle reentry, progression and differentiation in mammalian inner ear, maintaining the cell cycle arrest[4-7]. However, it has been reported that supporting cell proliferation and hair cell regeneration spontaneously occurs *in vitro *after aminoglycoside ototoxicity in the vestibular sensory epithelia of adult mammals, including guinea pigs and humans[8,9]. In these instances, new hair cells seem to originate from supporting cells that reenter the cell cycle and subsequently divide asymmetrically; or they may arise after transdifferentiation from supporting cells of the vestibular system, but not from cochlea[10,11].

It is now known that mouse adult vestibular sensory epithelia and neonatal organ of Corti tissue harbor cells that, when subjected to suspension culturing, are able to generate floating clonal colonies, the so-called spheres[12,13]. These spheres demonstrated capacity for self-renewal, and express inner ear precursor markers such as nestin and Sox2[14]. However, the sphere formation ability of the dissociated mouse cochlea decreases during the second and third postnatal weeks, in a way substantially faster than the vestibular organ, which maintains its stem cell populations up to more advanced ages[13]. These findings suggest that in the organ of Corti the stem cell properties become limited along the development. Standardization of procedures for cell culturing and characterization is a major step toward the study of cochlea progenitor cell differentiation and the definition of strategies for inner ear molecular, gene and cell therapy[15]. However, the establishment of dissociated organ of Corti suspension culture is still challenging. Although the guinea pig has been widely adopted as an animal model for cochlea experimental surgery[16], it has not been demonstrated as an appropriate source of cells for suspension culturing of the organ of Corti. The aim of this study was to compare conditions and outcomes of suspension cultures of dissociated organ of Corti from neonatal mouse and guinea pig, and to evaluate the guinea pig as a potential cochlea donor for preclinical cell therapy.

## Methods

The experimental protocol was previously approved by the Internal Review Board on Ethics in Animal Research from the Medical School and the Institute of Biosciences of the University of São Paulo. All experiments were conducted in accordance with the guidelines for the care and use of laboratory animals established by the American National Research Council[17]. In this study, we used postnatal day 3 (P3) C57BL/6J mouse (*Mus musculus*) and guinea pig (*Cavea porcellus*), obtained from specialized breeders (Biotério de Camundongos Isogênicos do Instituto de Ciências Biomédicas, USP and Centro de Desenvolvimento de Modelos Experimentais para Medicina e Biologia, CEDEME, UNIFESP, São Paulo, Brazil). Animals presenting acute or chronic ear infection or congenital malformations were excluded from the study. Animals were sacrificed in a carbon dioxide chamber.

### Tissue isolation and dissociation

After bathing the animals in absolute ethanol, they were decapitated and had the temporal bones removed and maintained in Leibovitz's L-15 medium (Sigma-Aldrich, St Louis MO). Cochlear sensory epithelia containing the organ of Corti were surgically isolated using micro-mechanical dissection technique under a stereo-microscope (Tecnival, SQF-F); *stria vascularis *and spiral ganglion were removed. The epithelia containing the organ of Corti were isolated, transferred to a flask containing 1 mL of HBSS solution (*Hank's Balanced Salt Solution*, 137 mM NaCl, 5.4 mM KCl, 0.3 mM Na_2_HPO_4_, 0.4 mM KH_2_PO_4_, 4.2 mM NaHCO_3_, 5.6 mM glucose, 300 mM HEPES pH 7.4) and 0.05 U/mL elastase (Sigma-Aldrich, St Louis MO), and incubated for 15 minutes at 37°C. Further enzymatic dissociation of organ of Corti was achieved by adding CaCl_2 _to 3 mM and 600 U/mL collagenase type II (Invitrogen, Carlsbad CA) and incubating for extra 15 minutes at 37°C. Trypsin dissociation of tissue was sequentially performed with 0.05% Tryple (Invitrogen, Carlsbad CA) for 15 min. at 37°C. Tissue was precipitated by gravity within the microtube, and the supernatant was discarded by aspiration. After washing the sample twice with HBSS, cells were mechanically dissociated by passing through fire-polished Pasteur pipettes with decreasing calibers and filtered through a 100-μm cell strainer (BD Falcon™) to remove cell debris. Twenty μL of the supernatant were used for cell morphology observation and counting at an Axiovert 40C microscope (Zeiss, Germany). Cell suspension was centrifuged at 200 × *g*, 4°C, for five minutes. The supernatant was discarded and the cells were resuspended in complete medium.

### Suspension cell culture of dissociated organ of Corti

To obtain suspension cultures, 10^4 ^cells were plated into a well of a 96-well dish previously coated with poly-HEME (Sigma-Aldrich, St Louis MO) to prevent cell attachment[18]. Cultures were maintained in a defined medium composed of DMEM-F12 (1:1), supplemented with 1X B27, 1X N2, 1X glutamine, 1X insulin, transferrin and selenium (ITS, all from Invitrogen, Carlsbad CA), ampicillin at 0,3 μg/mL (Teuto Brazilian Laboratory, Brazil), 20 ng/mL human epidermal growth factor (EGF), and either 10 ng/mL basic fibroblast growth factor (bFGF) or 20 ng/mL transforming growth factor alpha (TGFα, Invitrogen), at 37°C and 5% CO_2_. Fifty percent of the culture medium was replaced every 48 hours[19].

### Establishment of subcultures

The primary sphere cultures were maintained for seven days *in vitro *(DIV); while for first (P1) and second (P2) passages cells were cultured for five and three DIV, respectively. Passages were performed by adding Tryple (Invitrogen) to each well at a ratio of 1:1, at 37°C and 5% CO_2,_, for ten minutes, followed by mechanical dissociation with Pasteur pipettes. After spinning the cell suspension at 200 × *g*, 4°C, for four minutes, cells were resuspended with complete medium, counted, and plated at 10^4 ^cells per well.

### Otosphere differentiation

For analysis of cell differentiation, otospheres were transferred into poly-L-ornithine (0.1 mg/mL) and fibronectin (5 ug/mL) treated eight-well culture slides (BD Falcon™) and allowed to attach for 24 hours in wells filled with defined medium without growth factors. After the cells were attached, we replaced eighty percent of the medium DMEM-F12 (1:1) and repeated this procedure every second or third day. Differentiated cells were analyzed after seven DIV by indirect immunofluorescence.

### Indirect immunofluorescence and phenotypic sphere characterization

For sphere analyses and characterization by indirect immunofluorescence, P1 or P2 cells were transferred to coverslips within wells of a 24-well dish, previously coated with 30 μg/mL poly-D-lysine (Sigma) and 2 μg/mL laminin (Sigma). After plating, dishes were maintained for two hours, at 37°C and 5% CO_2, _and centrifuged at 200 × *g*, at 4°C, for two minutes[20]. The remaining medium was removed and sphere attachment to the coverslips was monitored microscopically. Cells were fixed in 4% paraformaldehide in HBSS for one hour at 37°C, rinsed in HBSS, and permeabilized in 0,3% triton X-100 for 20 minutes at room temperature. Cells were blocked in 10% goat serum (Santa Cruz Biotechnologies, Santa Cruz CA) and incubated with primary antibodies diluted in 3% bovine serum albumin (BSA, Invitrogen) in HBSS, for one hour at room temperature. Primary antibody dilutions were 1:100 for monoclonal anti-nestin (Chemicon), 1:100 for monoclonal anti-sox2 (Chemicon) or 1:50 for polyclonal anti-sox2 (Santa Cruz), 1:50 for polyclonal anti-myosinVIIa (Affinity BioReagents, ABR), 1:50 for polyclonal anti-jagged1 (Santa Cruz), 1:50 for monoclonal anti-p27kip1 (Abcam), 1:50 for polyclonal anti-jagged2 (Santa Cruz). Cells were rinsed in HBSS and incubated with secondary antibodies, diluted in HBSS-BSA, for one hour at room temperature: Cy3-conjugated anti-mouse (1:1000, Invitrogen), Alexa Fluor 488-conjugated anti-mouse, anti-goat and anti-rabbit (1:400, Invitrogen), Alexa Fluor 546-conjugated anti-goat and anti-rabbit (1:400, Invitrogen). Samples were mounted in ProLong Gold Antifade reagent (Invitrogen) containing DAPI (4',6-diamidine-2-phenyl indol) for nuclear identification. Images were acquired by fluorescence microscopy (Axioplan, Carl Zeiss, Germany) using a software to collect digital images (Isis Fish Imaging Meta System), and confocal microscopy (LSM410 or LSM510, Carl Zeiss, Germany), as indicated.

### Study groups and variables

Mouse and guinea pig organ of Corti suspension cultures were maintained overall for 15 DIV with EGF, and either bFGF or TGFα, for initial comparative analyses. Quantitative analysis was performed through direct counting the spheres from 20 consecutive microscope fields for each coverslip. For each growth factor treatment, bFGF or TGFα, two variables were examined: the number of spheres per coverslip and the number of cells in each sphere, each of them determined by confocal counting of DAPI-positive nuclei. These variables were compared between mouse and guinea pig cultures. We also observed the overall distribution of nestin and sox2.

### Statistical Analysis

The results were expressed as the mean ± standard deviation of the percentage of labeled cells in each growth factor treatment condition, EGF plus bFGF or EGF plus TGFα. The continuous variables were compared by Student's *t*-test. The level of statistical significance was set at p ≤ 0.05. Statistical analysis was performed using the GraphPad Instat program.

## Results

**Figure 1:**
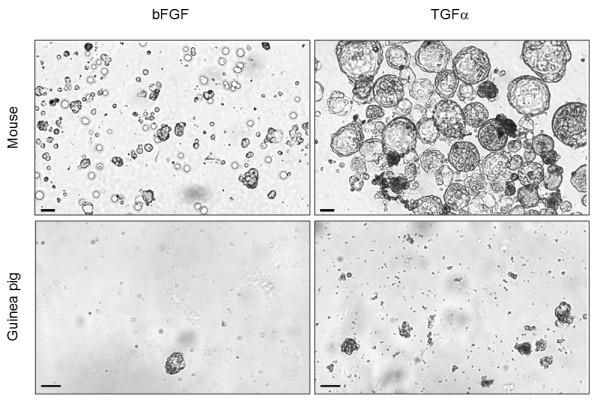
**Images represent analyses taken at a Zeiss Axiovert 40C inverted microscope and an Axiocamera MRC5 (Zeiss, Germany) of spheres observed with phase contrast while culturing of dissociated mouse or guinea pig cochleas, with either bFGF or TGFα, as indicated**. Scale bar 50 μm.

The most appropriate growth factor combination to provide a synergistic effect suitable for sphere formation is still a matter of research. Our choice was to use epidermal growth factor (EGF) in combination with either basic fibroblast growth factor (bFGF) or transforming growth factor alpha (TGFα), according to previous results from the literature[21]. We used dissociated mouse or guinea pig organ of Corti at postnatal day three (P3) in suspension cultures to compare the above conditions. We found a significant difference between groups regarding the number of sphere when data was combined for both animals, with more spheres observed in the TGFα group (23.3 ± 8.5) than in the bFGF group (9 ± 1, *p *= 0.044, Student's *t*-test). In addition, the TGFα group (37.6 ± 23.5) tended to present more cells in each sphere than the bFGF group although this comparison did not reach statistical significance (16.3 ± 4.1, *p *= 0.098, Student's *t*-test, Figure 1 and Table 1).

**Table 1:** Comparison of otosphere size parameters between treatment groups and species

Groups	EGF + bFGF	EGF + TGFα	***p***	Mouse*	Guinea pig*	***p***
Number of spheres per coverslip	9 ± 1	23.3 ± 8.5	0.044	18.5 ± 11	11.5 ± 4.9	0.458

Number of cells in each sphere	16.3 ± 4.1	37.6 ± 23.5	0.098	32.6 ± 30.5	12.5 ± 5.8	0.041

Values represent the mean ± 1 standard deviation; *p *is from Student's t-test; and * considers both growth factor treatment together.

When we analyzed the sphere number between organisms, we observed no difference in sphere number between mouse (18.5 ± 11) and guinea pig (11.5 ± 4.9) cultures (*p *= 0.458, *Student's t*-test). On the other hand, mouse cultures (32.6 ± 30.5) yielded a higher number of cells per spheres than guinea pig cultures (12.5 ± 5.8, *p *= 0.041, Student's *t*-test). We concluded therefore that TGFα in the presence of EGF increases the number of spheres in cultures of dissociated organ of Corti, when compared to bFGF. Our data also shows that at the neonatal period mouse cochlea yields more cells per sphere than the guinea pig ones.

**Figure 2:**
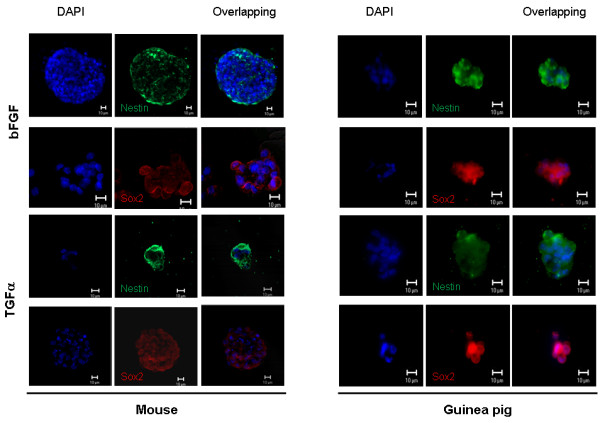
**Indirect immunofluorescence of mouse or guinea pig otospheres from first or second passage, cultivated in the presence of either bFGF or TGFα, as indicated**. The neural stem cell markers, sox2 and nestin, were used to label the cells. DAPI identifies cell nuclei. Scale bar 10 μm.

We analyzed the expression of two markers in the otospheres, nestin and sox2. The former is an intermediate filament expressed in neuroepithelial stem cells, during embryogenesis, employed as a marker of immature neurons and neuroblasts[22]. Sox2 is a transcription factor involved in sensory inner ear development, cell fate determination and stem cell maintenance. In cultures from both species, we detected sox2-positive and nestin-positive cells in all spheres analyzed, in a cytoplasmic distribution in roughly 40% of cells (Figure 2, arrows). Therefore, comparing mouse and guinea pig, we may consider that cochlea from both organisms yielded approximate numbers of spheres containing cells expressing markers of pluripotency.

**Figure 3:**
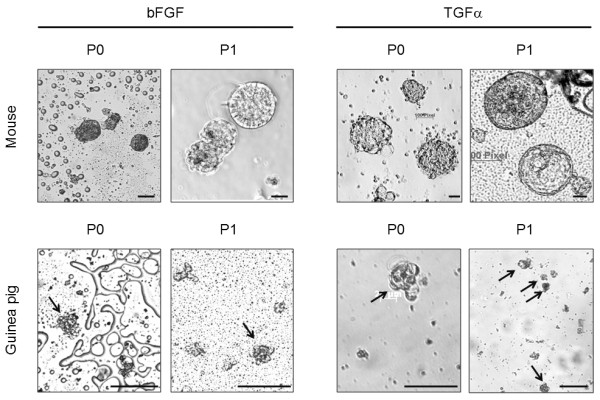
**Images of analyses taken at a Zeiss Axiovert 40C inverted microscope and an Axiocamera MRC5 (Zeiss, Germany) of spheres observed with phase contrast while culturing of dissociated mouse or guinea pig cochleas, with either bFGF or TGFα, as indicated**. P0 and P1 indicate primary culture and first subculturing, respectively. Arrows indicate otospheres obtained from guinea pig. Scale bar 50 μm.

**Figure 4:**
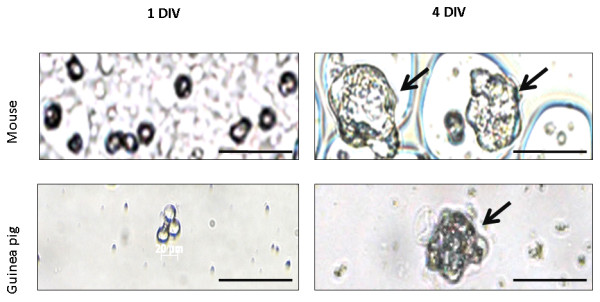
**Images represent analyses taken at a Zeiss Axiovert 40C inverted microscope and an Axiocamera MRC5 (Zeiss, Germany) of spheres observed with phase contrast while culturing of dissociated mouse or guinea pig cochleas, as indicated**. Data shown was obtained with TGFα-supplemented medium. Similarly, otospheres cultivated in culture medium with bFGF presented the same pattern of self-renewal (not shown). All images are from passage-one cells, cultivated for one (1DIV) or four (4DIV) days *in vitro*. Arrows indicate otospheres. Scale bar 50 μm.

We further investigated other stem/progenitor cell properties in the otospheres, such as self-renewal, proliferation and differentiation. As observed in Figure 3, passage of the primary cultures successfully yielded novel spheres. On the first day after subculturing cells were isolated or within floating colonies of two or three cells. Three days later, they had independently established multicellular floating colonies, otospheres (Figure 4). These are indirect evidences supporting the ability of those cells for self-renewal and proliferation, as the increasing size of otosphere along culturing time (Figure 3) suggests that cells dissociated from otospheres at passage may proliferate and form new otospheres.

Conditions for *in vitro *differentiation of otospheres into hair cells or supporting cells have been reported[12]. We cultured P1/P2 otospheres under adherent conditions in medium composition favoring differentiation into hair and supporting cells. We demonstrate the presence of cells expressing markers for either supporting (p27kip1 and jagged1) or hair cells (myoVIIa and jagged2) from mouse otospheres (Figure 4). As no adherence could be obtained for guinea pig otosphere, we could not observe cell differentiation. This may be explained by the low number of cells observed for guinea pig otosphere comparatively to the mouse.

## Discussion

Progenitor cells have been shown to be present in vertebrate sensory epithelia, based on a number of evidences: (1) sphere formation was demonstrated from inner ear sensory epithelia of birds[23,24], fish[25], neonatal rat cochlea[26], postnatal mouse cochlea and vestibular system[12,13], and adult human and guinea pig spiral ganglion[27]; (2) spheres were shown to be clonal and capable of self renewal[12,13]; and (3) spheres were able to differentiate into cell types corresponding to all three germ layers, ectoderm, endoderm, and mesoderm, indicating that these are pluripotent stem cells[28]. Cells in the spheres could differentiate into hair cells and neurons with inner ear cell properties[13,29]. This raises the possibility that, if properly stimulated, they can be induced to differentiate *in vivo *as the basis for future therapies, including replacement of cells in the inner ear[28].

More recent data from mammals suggests that supporting cells or a subset of supporting cells can act as precursors for hair cells, and several studies suggest that supporting cells have stem cell characteristics. Those properties may vary among the different supporting cell types, which have distinct morphologies and gene expression profiles[14,18,30,31]. Stem cell markers such as Sox2, Nestin, Musahi, Notch, Prox1, Islet1 were demonstrated to be expressed in postnatal supporting cells[32-37].

Nestin is an intermediate filament protein expressed by stem and progenitor cells early in development, and throughout the early postnatal period in the central and peripheral nervous systems, being considered a neural stem cell marker. It has been previously described in the organ of Corti of both developing and mature cochlea, suggesting the presence of immature precursor cells in the inner ear[14,33,38]. Nestin-positive cells expanded in culture from proliferating and floating spherical colonies have been shown to incorporate bromodesoyuridine into the DNA indicating their proliferation ability. In addition, they retain the ability to differentiate into cells displaying morphological features and expression of markers of hair cells and supporting cells[14,39]. Sox2, a transcription factor, is another marker of the inner ear prosensory domain. In developing central and peripheral nervous systems, Sox2 expression is associated with progenitor and stem cell populations and with the sensory progenitors of the cochlea. Sox2 is widely expressed in the otocyst, but as the inner ear develops and proneural cells delaminate, its expression becomes restricted to prosensory domains[40]. In experiments using fluorescent activated cell sorting (FACS) for isolation and purification of inner ear progenitor cells, from embryonic and postnatal cochlea, it was demonstrated that this specific population expresses cochlear sensory precursor markers as Sox2 and Nestin, and can differentiate *in vitro *into cells expressing markers of hair cells and supporting cells *in vitro*[18,31].

Culturing organ of Corti progenitor cells under nonadherent conditions is challenging, because *in vitro *cell density and proliferation are low. Several growth factors may promote the proliferation of vestibular sensory epithelial cells after damage, including EGF, bFGF, TGFα, insulin-like growth factor 1 (IGF1), and others[41-43]. A nonadherent culture typical for mouse organ of Corti, established at postnatal day three, with approximately 10^4 ^cells at seeding, contains 4 ± 2.08 spheres after six DIV without further growth factor supplementation[21,44]. According to Zine *et al*, after six DIV there were significantly more spheres formed, 41.25 ± 3.50 spheres, when the same amount of dissociated cells was maintained in EGF plus TGFα supplemented medium[21]. After the sixth DIV 50% of sphere cells presented Abcg2 staining, an epithelial progenitor cell marker[21]. The effects of these two growth factors on sphere formation are consistent with the results of our experiments, and with previous studies that have implicated the EGF and TGFα growth factor family in *in vitro *proliferation within sensory regions of mature utricles and organ of Corti explants[43-45]. Li *et al *observed that a combination of EGF plus IGF1 had a partially addictive effect resulting in a higher incidence of sphere formation, 68 ± 24 spheres per 10^5 ^plated cells, compared with single supplements, either EGF, bFGF or IGF1 alone, which provided 40 spheres per 10^5 ^plated cells[12]. Kuntz and Oesterle showed through autoradiographic techniques after tritiated thymidine labeling that simultaneous infusion of TGFα and insulin directly into the inner ear of adult rats stimulated DNA synthesis in the vestibular sensory receptor epithelium, with the production of new supporting cells and putative hair cells; however the infusion of insulin alone or TGFα alone failed to stimulate significant DNA synthesis[43]. Yamashita and Oesterle tested the effects of several growth factors on progenitor cell division in cultured mouse vestibular sensory epithelia and observed that cell proliferation was induced by TGFα in a dose-dependent manner, and by EGF when supplemented with insulin, but not by EGF alone[45]. Zheng et al examined the possible influence of 30 growth factors on the proliferation of rat utricular epithelial cells in culture and found that IGF1, TGFα and EGF stimulated cell proliferation[41]. Our experiments show that culture medium supplemented with TGFα has an additional effect on the number of forming spheres, 2.5 times higher when compared with bFGF group, in agreement with some observations of other authors. No significant difference was observed on cells numbers per sphere; however, there was a tendency toward higher values in the TGFα group. We were unable to demonstrate direct proliferative activity by BrdU labeling due to unspecific signals in immunofluorescence assays (data not shown). On the other hand, we registered during the culturing period the size expansion of otospheres from both organisms, which is suggestive of cell proliferation (Figure 3). In conclusion, our findings suggest that the combination of EGF and TGFα in the culture medium is a good alternative for otosphere production due to its higher rate of sphere formation.

**Figure 5:**
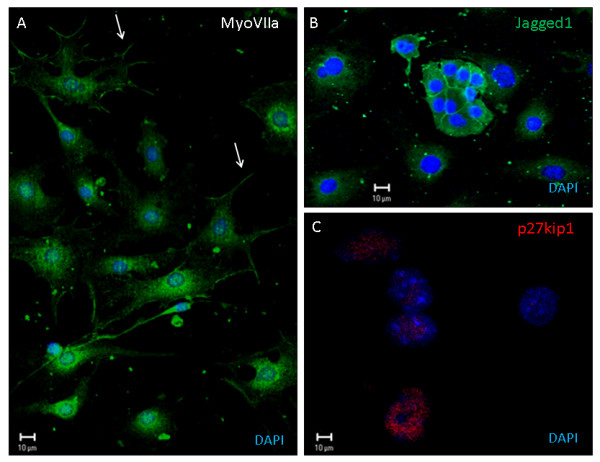
**Indirect immunofluorescence of mouse otospheres from second passage, cultivated in the presence of bFGF, and submitted to dish adherence and cell differentiation**. Myosin VIIa, a marker for hair cells, is labeled by Alexa 488 and shown in panel A. Arrows indicate plasma membrane processes, underneath which there is an enrichment of myosinVIIa. P27kip1 and Jagged 1, markers for supporting cells give the expected green staining of plasma membrane and red labeling of nuclei, respectively, shown in panels B and C. DAPI stains in blue nuclear DNA. Scale bar 10 μm.

**Figure 6:**
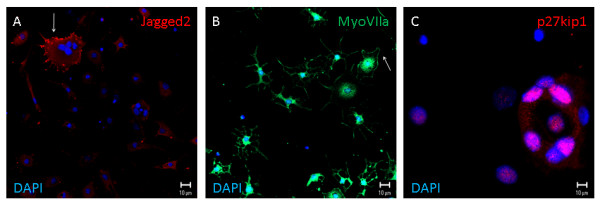
**Indirect immunofluorescence of mouse otospheres from second passage, cultivated in the presence of TGFα, and submitted to dish adherence and cell differentiation**. Jagged 2 (panel A) and Myosin VIIa (panel B) are hair cell markers, here labeled in red and green, respectively. Arrows indicate their concentration near the plasma membrane, especially in membrane ruffles. P27kip1 labels supporting cell nuclei, as shown in panel C. DAPI identifies the cell nucleus in blue. Scale bar 10 μm.

Dissociated guinea pig cochlea produced otospheres *in vitro*, expressing sox2 and nestin similarly to mouse otospheres. The presence of cells labeled for these two markers is supporting evidence for the presence of inner ear progenitor cells in the postnatal guinea pig, retaining an undifferentiated phenotype, as observed in the mouse. Our results clearly show the staining for protein markers for both hair cells and supporting cells upon culturing of mouse otospheres under conditions favoring cell differentiation (Figures 5 and 6). All markers employed, myosin VIIa and jagged2 for hair cells and p27kip1 and jagged1 for supporting cells, presented their expected subcellular distribution (myosinVIIa in cell processes, jagged 1 and 2 in the plasma membrane, and nuclear localization for p27kip1). This confirms the undifferentiated phenotype of the otospheres and its commitment to the cell types from the inner ear. We believe that the lack of demonstration of hair cell and supporting cell differentiation for guinea pig spheres is most probably due to their limited cell number (Figure 1 and Table 1). It may also be related to the relatively earlier maturation of guinea pig cochlea, which has been studied before. Comparisons between fetal and neonatal guinea pigs revealed that cochlear microphonics and endocochlear potential may be recorded in the prenatal period and reach adult levels at birth[46]. It has also been described that maturation of marginal cell junctions in guinea pigs occurs during the first few postnatal days, along with postnatal morphologic maturation of the organ of Corti and the *stria *vascularis, approximately one week after birth[47,48]. In mice, evoked potentials are compatible with hearing at 12 days after birth, while auditory maturation of guinea pig should occur 12-15 days before birth[49]. Oshima *et al *obtained few cells with potential to form spheres in the organ of Corti of 21-day-old mice, corresponding to nine days after the maturation of the auditory pathway[13]. As P3 guinea pigs should have had auditory maturation 15 days before, cells with sphere-forming ability may indeed be found. If the major drawback is their limited number, it is worth pursuing the best growth factor combination that potentially leads to increased cell survival, proliferation and differentiation. It may be likely, however, that a very small number of guinea pig cochlea progenitors impairs their viability *in vitro*. On the one hand, the cell viability, though partial, that we report here for P3 guinea pig cochlea progenitors reinforces this organism as an experimental animal model in studies searching for the mechanisms for organ of Corti regeneration. On the other hand, the limited sphere cell number and restricted differentiation potential observed by us for guinea pigs are evidences of their earlier cochlear maturation when compared to mouse.

## Conclusions

Dissociated guinea pig cochlea produced otospheres *in vitro*, expressing sox2 and nestin similarly to mouse otospheres. Culture medium supplemented with EGF plus TGFα yielded a higher number of spheres than medium containing EGF plus bFGF for both animals. Compared to culturing of dissociated guinea pig organ of Corti, mouse cultures yielded a higher number of cells per sphere. This lower number of cells for guinea pig spheres may relate to its lack of differentiation in vitro, as opposed to the strong differentiation potential observed *in vitro *for mouse otospheres.

## Competing interests

The authors declare that they have no competing interests.

## Authors' contributions

JO: design of the study, literature review for standardization of cell cultures, reproducibility of cell cultures, immunofluorescence assays, statistical analyses. LCMBJ: literature review for standardization of cell cultures, reproducibility of cell cultures and subcultures, microscope image acquisition. ACB: reproducibility of cell cultures, immunofluorescence assays, microscope image acquisition. KL: immunofluorescence assays, microscope image edition. RCMN: design of the study, critical review of data and the manuscript, and provider of the laboratory structure and support for the project. LAH: technical supervision on cell culturing and immunofluorescence analyses, final image selection and edition, final review of the manuscript. RFB: design and coordination of the study.

## Funding

FAPESP (Fundação de Amparo à Pesquisa do Estado de São Paulo)

CNPQ (Conselho Nacional de Desenvolvimento Científico e Tecnológico)
